# Nutrient withdrawal rescues growth factor-deprived cells from mTOR-dependent damage  

**DOI:** 10.18632/aging.100183

**Published:** 2010-08-24

**Authors:** Emiliano Panieri, Gabriele Toietta, Marina Mele, Valentina Labate, Sofia Chiatamone Ranieri, Salvatore Fusco, Valentina Tesori, Annalisa Antonini, Giuseppe Maulucci, Marco De Spirito, Tommaso Galeotti, Giovambattista Pani

**Affiliations:** ^1^ Institute of General Pathology, Laboratory of Cell Signaling, Catholic University Medical School, Rome Italy; ^2^ Institute of Physics and Microscopy Core Facility, Catholic University Medical School, Rome Italy; ^3^ Vascular Pathology Laboratory, Istituto Dermopatico dell'Immacolata - IDI- IRCCS, Rome, Italy; * These authors contributed equally to this work

**Keywords:** mTOR, nutrients, cell death, growth factor withdrawal, endothelial cells, ageing

## Abstract

Deregulated nutrient signaling plays pivotal roles in body ageing and in diabetic complications; biochemical cascades linking energy dysmetabolism to cell damage and loss are still incompletely clarified, and novel molecular paradigms and pharmacological targets critically needed. We provide evidence that in the retrovirus-packaging cell line HEK293-T Phoenix, massive cell death in serum-free medium is remarkably prevented or attenuated by either glucose or aminoacid withdrawal, and by the glycolysis inhibitor 2-deoxy-glucose. A similar protection was also elicited by interference with mitochondrial function, clearly suggesting involvement of energy metabolism in increased cell survival. Oxidative stress did not account for nutrient toxicity on serum-starved cells. Instead, nutrient restriction was associated with reduced activity of the mTOR/S6 Kinase cascade. Moreover, pharmacological and genetic manipulation of the mTOR pathway modulated in an opposite fashion signaling to S6K/S6 and cell viability in nutrient-repleted medium. Additionally, stimulation of the AMP-activated Protein Kinase concomitantly inhibited mTOR signaling and cell death, while neither event was affected by overexpression of the NAD+ dependent deacetylase Sirt-1, another cellular sensor of nutrient scarcity. Finally, blockade of the mTOR cascade reduced hyperglycemic damage also in a more pathophysiologically relevant model, i.e. in human umbilical vein endothelial cells (HUVEC) exposed to hyperglycemia. Taken together these findings point to a key role of the mTOR/S6K cascade in cell damage by excess nutrients and scarcity of growth-factors, a condition shared by diabetes and other ageing-related pathologies.

## Introduction

Mammalian cells sense availability of nutrients through a complex array of both paracrine/endocrine and cell-autonomous signaling cascades which regulate proliferation, differentiation and survival. Deregulated function of these cascades either due to nutrient excess or abnormal cell responses, play a central role in metabolic diseases such as diabetes and its complications [1,2], in body ageing 3] and cancer [4,5]. A better understanding of the molecular interactions underlying cellular consequences of exposure to energy substrates is therefore key to the understanding, the prevention and the therapy of severe and epidemiologically relevant human diseases.

The mTOR (mammalian Target of Rapamycin)/FRAP cascade serves a unique function in coordinating nutrient availability and energy metabolism with cell response to growth factors [6,7]. By phosphorylating and activating the S6 kinase or inhibiting the Elongation Factor 4 Binding Protein-1 (4EBP-1), mTOR stimulates the ribosomal translation of different classes of mRNAs, thereby promoting protein synthesis. It also acts directly on gene expression by phosphorylating transcription factors mainly involved in the orchestration of glucose and lipid metabolism [7]. Accordingly, mTOR activity is exquisitely sensitive to cell energy status, sensed through a complex circuitry involving the AMP-activated kinase, a serine threonine kinase activated by the reduction of intracellular ATP and the increase of AMP/ATP ratio [8] Phosphorylation of the TSC1/TSC2 complex by AMPK and the consequent inactivation of the GTPase Rheb, an upstream activator of mTOR, profoundly inhibits mTOR signaling, thereby reducing protein synthesis and promoting cell survival under nutrient restriction [8]. mTOR is also directly regulated by aminoacids, through a distinct mechanism involving the GTPase Rag [9]. Finally, the mTOR cascade is crucial for signaling downstream of growth factor receptors including the insulin receptor. It is in fact, activated, in a TSC- and Rheb-dependent fashion, by growth factors through PI3 kinase and the serine-threonine kinase AkT/PKB [10].  Consequently, the mTOR cascade integrates nutritional and mitogenic/antiapoptotic cues ensuring that energy supply and protein synthesis are adequate to support cell growth (i.e. increase in cell size), proliferation, and accumulation of biomass.

Most of nutrient-related functions of mTOR are mediated by a multimolecular complex including mTOR itself and the scaffold protein Raptor (a complex indicated as TORC1) [11]. Nonetheless, additional mTOR signaling capacity directed towards AkT/PKB also involves a second, largely nutrient- and rapamycin-insensitive complex (TORC2) centered on Rictor as main scaffold component [12]. Thus, mTOR operates both upstream and downstream of PKB/AkT, revealing an intricate cross-talk with PKB-dependent survival and mitogenic signaling at the intersection between cell metabolism and regulation of normal tissue growth.

Hyperactivation of the mTOR/S6K axis has recently drawn significant attention as a key factor in the establishment of obesity and insulin resistance by nutrient overload [13]. S6K deficient mice display increased life span and resistance to age-related pathologies including loss of insulin sensitivity [14] Moreover, mTOR hyperactivation by excess nutrients negatively influences, both *in vivo* and *in vitro*, insulin and growth/trophic factor signaling, through the feed-back inhibition of upstream components such as the Insulin receptor Substrate 1 (IRS-1) [13, 15-17]. Finally, it has been demonstrated that mTOR activation leads to cell senescence in the context of block of the cell cycle [18], and, more in general, evidence exist that the mTOR cascade may play a central role in the signaling derangement that underlies tissue and body ageing [19].

Hence, converging lines of evidence indicate that mTOR and its downstream pathway, by transducing nutrient-triggered signals, may mediate cellular damage, through molecular mechanisms largely involving mTOR cross-talk with growth factor-triggered mitogenic and survival cascades.

Here we report a novel mechanism for cell survival regulation by nutrients. In particular, our findings reveal that unbalanced mTOR activity in the absence of adequate growth factor supply, may represent a general mechanism of cell death by excess nutrients. This may be relevant in the study of tissue hyperglycemic damage, in body senescence and cancer therapy, prospectively suggesting a possible pharmacological target for novel preventive and therapeutic strategies.

## Results

### Nutrient restriction protects 293T *Phoenix* cells from death by serum deprivation

**Figure 1.**
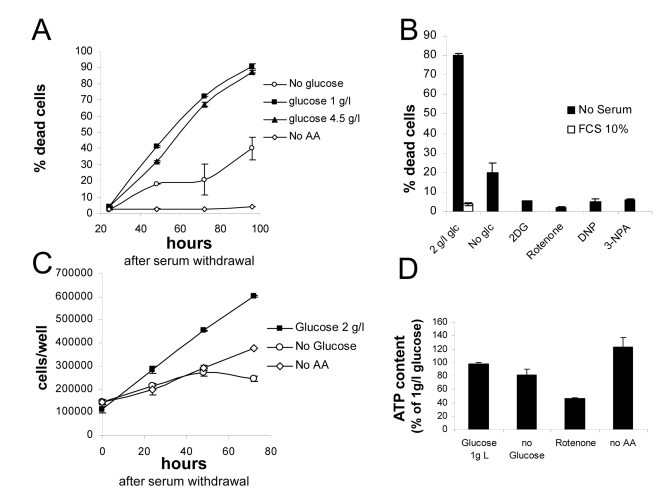
(**A**) Survival assay displaying progressive loss viability of nutrient-repleted *Phoenix* cells in serum free medium, and protection by either glucose or aminoacid deprivation. Values are Mean±SD of triplicate samples from one of several independent experiments. (**B**) Effect of metabolic inhibitors on cell death by serum deprivation in nutrient-rich medium. Death in the presence of serum was marginal, not affected by inhibitors and is therefore displayed only for the 2 g/l glucose sample. Extent of cell death in the absence of glucose is also reported. Values are Mean±SD of triplicate samples. Panel representative of several independent experiments with very similar results. (**C**) Growth curves for *Phoenix* cells grown in the absence of serum with or without nutrients. Numbers refer to live cells, based on morphological features and trypan blue exclusion. Values are Mean±SD of triplicate samples. Panel representative of two independent experiments. (**D**) Determination of ATP content in cells incubated for 24 hours in the indicated conditions. Values are % of the control (1 g/l glucose + aminoacids) sample. Chemiluminescence values were normalized for protein content of the different samples. Representative of two independent experiments.

Most immortalized cell lines undergo mitotic catastrophe and cell death with morphological and biochemical features of apoptosis when deprived of fetal calf serum or growth factor supply [20]. Upon serum withdrawal, 293-T *Phoenix *cells, a retrovirus packaging line derived from E1A-transformed embryonic human kidney cells (HEK-293) carrying a temperature sensitive T antigen, displayed severe and time-dependent loss of viability, as revealed by a Propidium Iodide uptake assay (Figure 1A). Nearly 100% of cells appeared dead by day 4 of culture (96 hours) (Figure 1A). Remarkably, removal from the culture medium of either Glucose or Aminoacid Supplement (Glutamine + DMEM Non Essential Aminoacids), the two main energy fuels for most cultured transformed cells [21], resulted in a drastic protection from cell death. Typically we detected a maximum of mortality of up to 30% at day 4 under glucose deprivation, and below 10%, comparable to average mortality in the presence of serum (Figure 1B), for aminoacid-starved cultures.Reduction of glucose from high (4.5 g/l) to low (1 g/l) concentration had no significant effect on cell viability, indicating that even physiological concentrations of glucose promote death of *Phoenix* cells in the absence of serum. Simultaneousremoval of glucose and aminoacid supplement from the culture medium resulted in rapid (12 hours) loss of viability, in a fashion which could not be prevented by addition of Pyruvate, Dimethyl-Succinate or Free Fatty Acids (not shown); this confirms that glucose and glutamine account for most of the energy supply for these cells, at least in the tested experimental conditions.

Live cell count revealed that *Phoenix* cells continue proliferating robustly in the absence of serum, and are therefore, at least in part, self-sufficient for mitogenic stimulation. Cell proliferation and death appear to occur concomitantly (Figures 1A and 1C), and are likely to be mechanistically linked [22]. Proliferation also occurred, although to a lesser extent, in nutrient deprived cultures, yet associated with no or minimal cell loss (Figures 1A and 1C).

Beneficial effect of nutrient restriction on cell viability prompted us to evaluate the consequence of pharmacological interference with cellular metabolism. As expected, the glycolysis inhibitor 2-deoxyglucose fully rescued cells from death in the presence of glucose, to an even larger extent than glucose deprivation (Figure 1B). Similarly, significant protection was obtained by interference with mitochondrial respiration: in fact, both complex I inhibitor Rotenone and complex II inhibitor 3-Nitropropionic acid (NPA) drastically reduced death of serum-deprived cultures. Also the uncoupling agent 2,4-dinitrophenol(2,4-DNP), at non toxic concentration, had the same protective effect as mitochondrial inhibitors on cell survival in 2 g/l glucose (Figure 1B); noteworthy, both DNP and electron transport chain (ETC) blockers rapidly killed *Phoenix* cells in the absence of glucose (not shown), indicating that mitochondria are functional in this cell line and support energy demand when glycolysis is prevented.

In order to evaluate the impact of nutrient restriction on the energy balance of *Phoenix* cells, ATP content was measured 48 hours after cell transfer to the different culture media. As expected based on survival data, no drastic reductions in cellular ATP levels were observed upon nutrient withdrawal (Figure 1D). Glucose deprivation led to a modest (about 20%) decrease of cellular ATP, and aminoacid removal to no reduction at all, compared to standard growth medium (2 g/l glucose and aminoacid supplement). ATP reduction was more pronounced (about 50%) in cells treated with Rotenone (Figure 1D), indicating that mitochondria contribute significantly to ATP generation in this tumor cell line.

Thus, survival of *Phoenix* cells in serum free medium is clearly subdued to a metabolic regulation by nutrient availability, that operates independently from severe changes in cellular energy levels.

### Nutrient toxicity in serum-deprived Phoenix cells is not mediated by ROS

**Figure 2.**
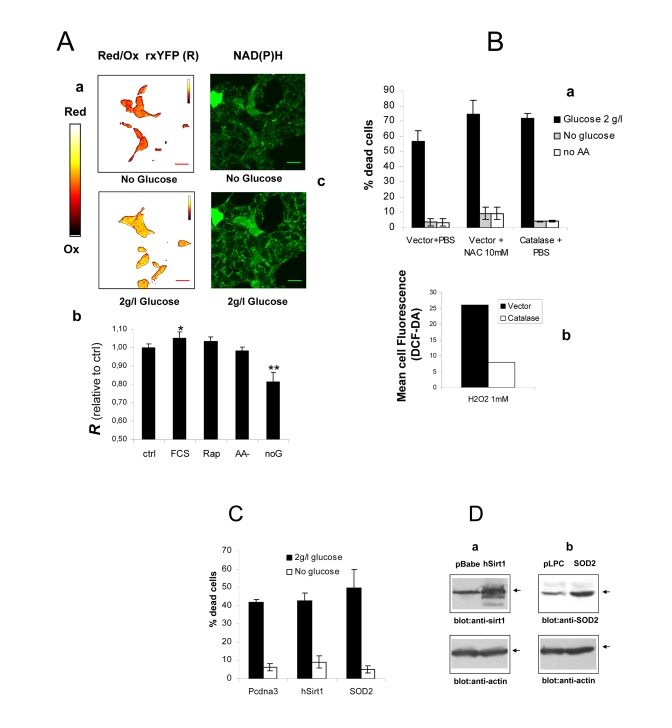
(**A**) ***a*** Intracellular redox state under nutrient restriction. ***a*** pseudocolor image (color bar on the left) of *Phoenix* cells expressing a redox-sensitive variant of the Yellow Fluorescent Protein (rxYFP) after 24 hours incubation in the absence of glucose (upper) and 30 minutes glucose re-feeding, in serum-free medium. Color shift from red to yellow indicates reduction of the fluorescent sensor. ***b*** Quantitation of mean R values over several regions of interest is reported. Data sets were compared by two-tailed t-test for independent samples. ***c*** Microfluorimetric analysis of reduced intracellular reduced NAD(P)H, based on cell green autofluorescence. Cells were excited in the two-photon mode at 366 nm and autofluorescence collected between 380 and 550 nm. Increase in cell brightness in the glucose-fed samples indicates accumulation of reduced pyridine nucleotides. (**B**) ***a*** Effect of antioxidants Catalase and N-Acetyl-Cysteine on cell viability in the presence and absence of glucose. Cells were transfected with a construct encoding human Catalase or the corresponding empty vector 48 hours before nutrient and serum starvation. Mock-transfected cells were also treated with 10 mM NAC as an alternative ROS scavenger. Values are mean±SD of triplicate wells. The experiment was repeated twice with identical results. ***b*** Cytofluorimetric analysis of cells loaded with the redox sensitive die H2-DCF-DA and exposed to a bolus (1 mM) of extracellular Hydrogen Peroxide. Decreased oxidation in the Catalase-transfected samples confirms elevated H2O2 degrading capacity in these cells. (**C**) Lack of effect of the longevity protein Sirt1 and the mitochondrial superoxide scavenger SOD2 on *Phoenix* cell viability in the presence of glucose and under glucose deprivation. Cell viability was scored at 72 hours after cell starvation. Representative of two comparable experiments. (**D**) Western blot analysis of Sirt1 (***a***) and SOD2 (***b***) expression in transfected cells. Transfection efficiency was normally around 50% based on expression of GFP.

Cell death by serum withdrawal is associated with the formation of harmful reactive oxygen species (ROS) [20], and nutrients may generate ROS through their oxidation in mitochondria [23]. Since nutrient restriction or mitochondrial blockade rescued *Phoenix* cells from serum deprivation, we tested possibility that cell protection might be mediated by an attenuation of cellular oxidative stress. To this end, *Phoenix* cells were transiently transfected with a redox-sensitive variant of the yellow fluorescent protein (rxYFP) and the intracellular redox state evaluated by confocal microscopy and fluorescence ratiometric analysis, 24 hours after serum or serum and glucose deprivation. RxYFP consistently appeared more reduced (as indicated by higher values of the Ratiometric Index *R*) in glucose-fed than in glucose-starved cells, revealing significantly higher levels of ROS in the latter cell population (Figures 2A, a and b). This finding was further supported by evidence of higher content of reduced NAD(P)H in glucose-fed cultures, as determined by cell microfluorimetry (Figure 2A, c). No significant redox changes were observed in cells deprived of Glutamine and NEAA or exposed to the mTOR inhibitor Rapamycin. As expected, addition of FCS further reduced the intracellular environment in glucose-fed cells (Figure 2A, b).

Based on these findings, excess oxidative stress unlikely accounts for impaired cell viability by nutrients. In keeping with this conclusion, no major changes in cell viability were induced, in the presence or absence of glucose, by saturating concentration of the ROS scavenger and glutatione precursor N-acethyl-cysteine (NAC, 10 mM) (Figure 2B, a). Similarly, overexpression of the ROS scavengers Catalase (Figure 2B, a) and SOD2 (Figure 2C and 2D, b) did not provide glucose-fed cells protection from death, nor affected cell viability in glucose free-medium. Notably, overexpression of Catalase effectively increased cell antioxidant capacity, as revealed by flow cytometry of cells loaded with the redox-sensitive dye Dichlorofluoresceine Diacetate (H2-DCF-DA) and exposed to a bolus of exogenous hydrogen peroxide (Figure 2B, b). Finally, overexpression of the class III deacetylase Sirt-1, a molecule linking, in model organisms and in mammalian cells, nutrient restriction to increased resistance to oxidative stress [24], did not rescue cells from glucose-induced death in serum-free medium (Figures 2C and 2D, a). Thus, collectively, these data suggest that generation of ROS and oxidative stress do not mediate the effects of glucose on cell viability in our experimental model. Additionally, failure of Sirtuin-1 to prevent or attenuate glucose-induced cell death indicates that this major nutrient sensor and regulator of cell survival is unlikely involved in the protective response of *Phoenix* cells to nutrient restriction.

### Blockade of mTOR prevents nutrient-induced cell death

Since glucose and aminoacid withdrawal provided comparable protection to serum-starved *Phoenix* cells, in spite of having different effects on cell energy (Figure 1D) and redox balance (Figures 2A and B and data not shown), we reasoned that a common signaling mechanism might underlie the antiapoptotic action of the two starvation modes. The mTOR/S6K signaling cascade, which is modulated by both glucose and aminoacids and regulates cell proliferation and survival [6], was therefore evaluated as a potential candidate.

**Figure 3.**
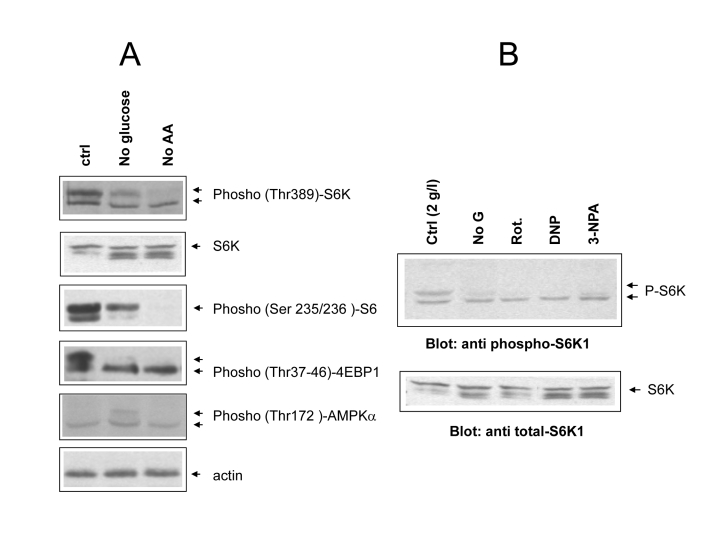
(**A**) Phospho-specific immunoblot analysis of mTOR/S6 kinase cascade activity under different cell feeding conditions. Cells were incubated for 24 hours in the indicated conditions (ctrl= 2g/l glucose + Aminoacids; noAA= glutamine and NEAA omitted). Where possible the same filter was cut into parallel strips and hybridized contemporarily with different antisera. When molecular weights of target proteins overlapped, filter were stripped and re-hybridized, or twin filters were prepared with the same protein lysates. Hyperphosphorylated protein species usually migrate slower and are indicated by separate arrows. Picture representative of several independent experiments. (**B**) Effect of metabolic inhibitors from figure 1B on S6 kinase phosphorylation. Upper arrows indicate the fully phosphorylated forms. Equal content of total S6 kinase in the different samples was verified by anti total S6K immunoblotting of the same protein lysates on a different nitrocellulose membrane. Picture representative of 2-3 three independent experiments.

Even in the absence of exogenous growth factors, mTOR activity remained remarkably elevated in glucose-fed cells 24 hours after serum withdrawal, as revealed by the phosphorylation patterns of the major mTOR effectors S6 kinase and 4E-BP1, and of the downstream substrate S6 (Figure 3A, lane 1). Note that in this analysis phospho-site specific antibodies often recognize multiple bands, the uppermost, slowest-migrating one generally representing the most heavily phosphorylated form of the protein (see arrows) [25]. Based on this criterion, we observed a marked reduction of mTOR activity in glucose-starved, and to an even larger extent, in aminoacid-starved cells (Figure 3A, lanes 2 and 3). A drastic reduction in S6 kinase phosphorylation was also observed in glucose-fed cells treated with mitochondrial inhibitors or with the uncoupler 2,4-DNP, in keeping with the starvation-mimicking effects of these treatments on cell survival (Figures 3B and 1C). In glucose-starved cells we also observed a small increase in the phosphorylation of the AMP-activated protein kinase (AMPK-α) (Figure 3A), the putative negative regulator of mTOR in this experimental condition [8]. This modest, although detectable biochemical change, which reflects the small reduction in cellular ATP content reported in figure 1 D, likely accounts for reduced mTOR signaling (Figure 1B) [8] in *Phoenix* cells grown in the absence of glucose. Thus, collectively, these observations confirm that mTOR is responsive to glucose and aminoacids, and that its activity positively correlates with nutrient availability and extent of cell death in serum-deprived *Phoenix* cells.

**Figure 4.**
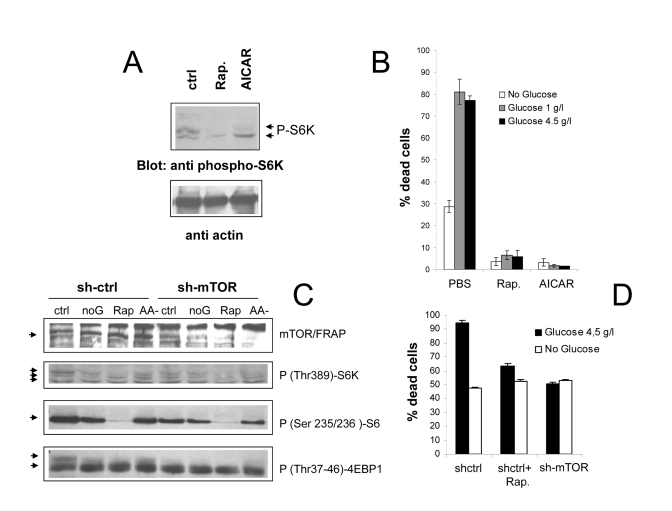
(**A**) Anti phospho S6K immunoblot analysis of *Phoenix* cells treated with the mTOR/FRAP inhibitor Rapamycin (200 nM) or the AMPK agonist AICAR (1 mM) for 24 hours in serum-free, nutrient rich medium. Ctrl=untreated cells.  A lower strip of the same filter was hybridized with anti-actin antiserum, to confirm equal protein loading. (**B**) Effect of pharmacological inhibition of the mTOR pathway on cell survival to serum deprivation under different feeding conditions. Values are mean±SD of triplicate samples. Representative of several independent experiments. (**C**) Immunoblot analysis demonstrating effective downregulation of mTOR/FRAP by lentiviral transduction of a targeting (sh-mTOR) or non-targeting (sh-ctrl) short hairpin RNA, and effects on the downstream signaling cascade. Cells were analyzed 24 hours after serum starvation in the indicated media (ctrl=2 g/l glucose + Aminoacids; noG= no Glucose; Rap= Rapamycin 200nM; AA- = 2 g/l glucose without glutamine and NEAA). In the anti p-S6K and anti p-4EBP1 a selective loss of the slow migrating, hyperphosphorylated band by nutrient-repleted sh-mTOR samples can be appreciated. (**D**) Survival assay displaying reduced mortality of sh-mTOR transduced *Phoenix* cells in serum-free, nutrient repleted medium. Note that nutrient-independent loss of viability was unusually high in these experimental conditions. Values are mean±SD of triplicate samples. Panel representative of two experiments performed with cells from two independent infections.

In order to address the role of the mTOR/S6K cascade in nutrient-dependent death of *Phoenix* cells, we evaluated the effect of mTOR blockade on cell viability in standard and glucose-depleted medium. Rapamycin, a macrolide antibiotic widely used as an immunosuppressive drug, directly inhibits mTOR activity within the nutrient sensitive TORC1 complex, by complexing with the cellular protein FKBP12; another drug, 5-aminoimidazole-4-carboxamide ribo- nucleoside (AICAR), indirectly suppresses mTOR signaling through AMP kinase, by mimicking cell de-energization and accumulation of adenosine mono-phosphate (AMP) [26]. As expected, both drugs drastically decreased the phosphorylation of the mTOR substrate S6 kinase in cells grown in the presence of both glucose and aminoacids (Figure 4A). More importantly, both Rapamycin and AICAR dramatically reduced cell death in nutrient repleted medium (Figure 4B).

In order to rule out potential non-specific effects of drug compounds, in a parallel series of experiments mTOR expression in *Phoenix* cells was genetically inactivated by shRNA technology. As displayed in figure 3C, lentiviral expression of a mTOR specific shRNA resulted in a substantial reduction of the mTOR expression level, and in a reduced phosphorylation of its downstream targets S6K, S6 and 4E-BP1, in nutrient-rich samples (Figure 4C, compare lanes 1 and 4 in each panel). In keeping with evidence of cell protection by Rapamycin and AICAR, mTOR inactivation allowed a higher percentage of cells (about 50%) to survive in nutrient repleted medium with respect to mock-infected cells, and nearly abolished protection by glucose withdrawal (Figure 4D). It should be noted, however, that here and in general in experiments involving genetic manipulation of *Phoenix* cells, mortality in the absence of nutrients was often higher than the usual (compare Figure 1A with 4D and S2,a), possibly due to cellular distress from the experimental procedure. Notwithstanding this limitation, survival data with mTOR-silenced cells confirm the observations made with chemical inhibitors, demonstrating that activation of the mTOR cascade is instrumental to nutrient-triggered cell death in the cell line under study, and that protection by nutrient restriction is conceivably mediated by the inhibition of this cascade.

**Figure 5.**
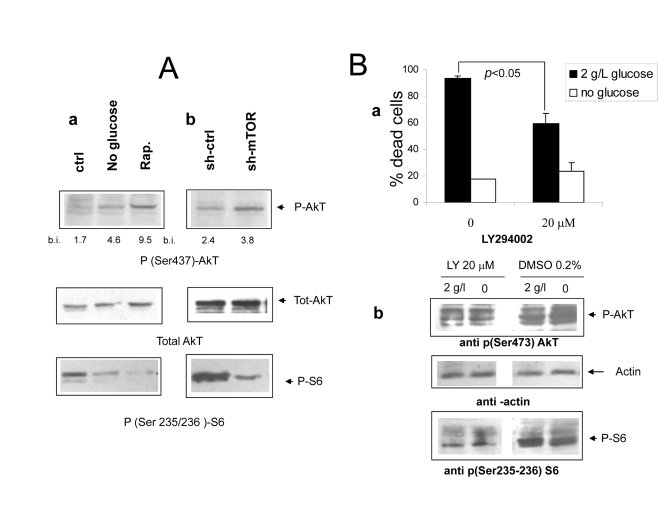
(**A** ***a*** Immunoblot analysis revealing increased phosphorylation of AkT/PKB on serine 473 under nutrient deprivation (upper panel). The relevant band is indicated by the arrow. Band quantization values (band volume) in band intensity (b.i.) units are indicated. The same filter was stripped and re-hybridized with an anti total AkT antiserum to ensure equal protein expression and sample loading (central panel); a lower strip of the same filter was hybridized with an antiserum specific for phospho S6 (lower panel, band indicated by arrow). Picture representative of several independent experiments. **b** Protein lysates from mock and mTOR-silenced cells grown under serum free DMEM with glucose and aminoacids were treated as in A. Relevant bands are indicated by arrows.  Densitometry of p-AkT bands is reported. (**B**) ***a*** Effect of the PI3 Kinase inhibitor compound LY294402 on *Phoenix* cell survival in serum-free medium. Cells were incubated for 72 hours with or without glucose as indicated. The inhibitor or vehicle alone (DMSO, 1:500 final dilution) were added at time 0. Values are Mean ±SD of triplicate wells. Representative of three independent experiments. Note that lower concentrations of LY294002 had no effect on cell survival in either medium. ***b*** Immunoblot analysis of protein lysates from cells treated as in a and incubated for 24 hours. Phospho-AkT (serine 473) and phospho-S6 (serine 235-236) were detected by specific antisera. Relevant bands (the middle one within the triplet for AkT) are indicated by arrows; equal protein loading was verified by reversible Ponceau S staining.

**Akt is activated by mTOR inhibition but does not account for cell protection **While in most cancer-related models the mTOR cascade exerts antiapoptotic functions downstream of the PI3 kinase/AkT PKB signaling axis [27], few examples of cell protection by inhibition of mTOR/S6K have been reported [28-31]. It is also known that hyperactivation of the mTOR cascade can downregulate survival signaling by the AkT/PKB kinase [15,16]. In search for a molecular mechanism linking nutrient-dependent mTOR signaling to massive cell death of serum-deprived *Phoenix* cells, we sought to evaluate the phosphorylation of AkT at serine 437, a biochemical correlate of AkT kinase activity. Consistent with previous reports, we found increased levels of AkT phosphorylation/activity in cells deprived of glucose or treated with Rapamycin, in a fashion which inversely correlated with activation of the mTOR effector S6 kinase (Figure 5 A, a). mTOR-silenced cells also displayed increased phosphorylation of AkT in nutrient rich medium, although to a lower extent compared to control cells treated with Rapamycin (Figures 5A, a and b); moreover, transfection of rat mTOR cDNA in cells deprived of human mTOR rescued mTOR expression and activity (as assessed by phosphorylation of S6) and in parallel decreased the phosphorylation of AkT (Supplementary Figure 1). Thus, taken together, these observations confirmed that, in serum-deprived *Phoenix* cells, nutrients downregulate AkT phosphorylation/activity through the mTOR cascade. This raises the possibility that increased AkT function might be responsible, at least in part, for the dramatic protection provided by restriction of glucose or aminoacid supply in this experimental model.

**Figure 6.**
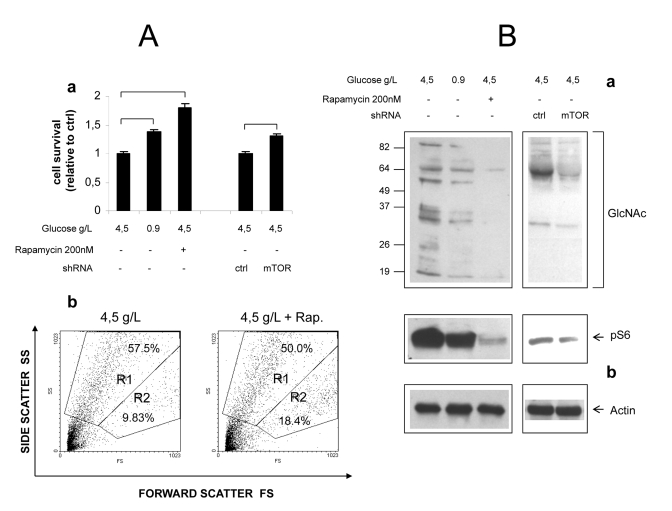
Role of mTOR in hyperglycemic damage of HUVEC cells. (**A**) ***a*** Effect of glucose, Rapamycin and mTOR knock-down on survival of growth factor-starved HUVECs.  Values are relative to cell survival in high glucose (10-15% survival). Numbers are mean± SD of four samples from two independent  experiments. All the indicated comparisons were significant by at least p<0.05 (two-tailed unpaired T-test). ***b*** Representative Forward/Side scatter plots of live (Region R2) and dead (Region R1) cells under high glucose and high glucose + Rapamycin. Raw numbers indicate percentages with respect to all the plotted events, including cell debris. Survivals were calculated on relevant regions only, according to the formula %survival=  %R2/(%R1+%R2). (**B**) Western blot analysis of GlcNAcylated proteins in total lysates of HUVEC cells. Glucose, Rapamycin and mTOR knock-down were combined as indicated. Impact of treatments on mTOR signaling was evaluated by anti phospho S6 immunoblotting (***b***). Equal protein loading was verified by anti-actin staining. Blots representative of two independent experiments.

To further investigate the mechanistic role of the PI3K-AkT cascade in cell survival induced by nutrient deprivation, cells were treated with the PI3 kinase specific inhibitor LY294002, and exposed to normal or glucose-deprived media in the absence of serum. Surprisingly, the PI3K inhibitor failed to reverse cell protection by glucose withdrawal, but attenuated cell death in nutrient-repleted medium (Figure 6A, a), suggesting that residual AkT activity may be detrimental rather than protective in this culture condition. Interestingly, biochemical studies revealed that, while AkT/PKB phosphorylation was, as expected, decreased, also the serine phosphorylation of S6 was strongly downregulated. This is an evidence that inhibition of the mTOR cascade, a downstream target of AkT [10], accompanied PI3K blockade (Figure 6A, b).

In a complementary series of experiments, over-expression of a constitutively active form of AkT (myrAkT) in *Phoenix* cells failed to prevent cell death in nutrient rich medium, while slightly decreasing cell protection by glutamine withdrawal (Supplementary Figure 2).

Collectively, these data do not support a role for AkT in cell survival by nutrient restriction in our cell model, but rather indicate that protection operates also in the context of PI3K (and AkT) inhibition, provided that the mTOR cascade is also blocked. Conversely, AkT appears to increase cell death in the presence of abundant nutrients, and to negatively interfere with cell protection by Glutamine deprivation.

### mTOR inhibition attenuates hyperglycemic damage in primary endothelial cells

In an attempt to verify that mTOR-dependent nutrient toxicity is not restricted to one single transformed cell line, we cultivated primary human endothelial cells (HUVEC) in high (4.5 g/L) ambient glucose, a well established model of endothelial hyperglycemic damage. Specific endothelial growth factors (EGF, FGF-B, VEGF and IGF-1), normally required for the optimal propagation of these cells, were omitted from the culture medium, while FBS (5%) was included to limit cellular stress. In these harsh conditions, a majority of cells detached from the plate and appeared dead after 48-60 hours of incubation, the percentage of live cells (quantified by flow cytometry as the percentage of cells with high forward scatter, low side scatter profile) ranging from 10 to 15%. Cell survival, however was significantly improved (nearly doubled) by Rapamycin, to an extent even larger than by cultivation in normal (0.9 g/L) glucose (Figure 6A, a). Importantly, these differences matched the phosphorylation level of S6, an index of mTOR activity (Figure 6B). Likewise, mTOR knock-down by lentivirus-delivered shRNA consistently increased cell survival by about 20%, in accordance with the evident although incomplete inhibitory effect on mTOR signaling (Figures 6A, a and 6B).

Additionally, accumulation of O-GlcNacylated proteins, a biochemical hallmark of endothelial damage by high glucose [2,32], was drastically reduced by Rapamycin and, although to a lesser extent, by mTOR knock-down (Figure 6B).

Thus, inhibition of the mTOR cascade partially rescues primary human endothelial cells from hyperglycemic damage under growth factor restriction, confirming and extending analogous findings obtained in *Phoenix *cells.

## Discussion

We describe here a novel mechanism for cell survival regulation by nutrients, our major conclusion being that activation of the mTOR signaling pathway is detrimental to cell survival in the context of growth factor scarcity. This conclusion is mainly based on mechanistic studies performed on a widely used tumor cell line, but has also been validated using a cell model (human primary endothelial cells) relevant to nutrient-related pathologies like vascular ageing and diabetic complications.

We have shown that, in the absence of exogenous growth factors, the 293-T "*Phoenix*" retrovirus packaging cell line undergoes massive cell death in a fashion strictly dependent on the availability of nutrients in the growth medium. In particular, with respect to a normally supplemented medium containing both glucose (either 4.5 g/l or 1 g/l), and glutamine + non essential aminoacids, withdrawal of either supplement exerts a remarkable protective effect with a nearly complete rescue of the culture, at least in the considered time frame (3-4 days). Importantly, although not investigated in detail, morphological data and flow cytometry evidence of subdiploid DNA accumulation and high side-scattering cell profiles (not shown) clearly suggest that nutrient-induced death of *Phoenix* cells largely occurs by apoptosis.

From a biochemical point of view, we have clearly demonstrated the involvement of the nutrient sensor mTOR in the protective cell response to nutrient restriction, and investigated its complex relation with the PI3 kinase/AkT signaling cascade. In view of the growing attention towards the mTOR/S6K cascade as a signaling module at the crossroad of multiple pathogenic mechanisms from diabetes and ageing to cancer, the observation that mTOR inhibition mediates the cell protective effect of nutrient withdrawal adds special value to our observation.

Cell damage by excess nutrient contributes to important pathologic conditions including Metabolic Syndrome, insulin resistance and diabetic micro- and macro-angiopatic complications. In a current model for hyperglycemic vascular damage, multiple pathogenic mechanisms (including deregulation of the polyol and hexosamine pathways and hyperactivation of PKC) are triggered in endothelial cells by glucose-driven overproduction of reactive oxygen species [2]. Our results significantly diverge from this model: first of all, death of *Phoenix* cells in high nutrients does not seem to involve Reactive Oxygen Species, although mitochondrial inhibitors, but not antioxidants, provide a significant protective effect. Second, not only hyperglycemia, but also physiological (5 mM) concentrations of glucose appear "toxic" in our model, in presence of glutamine/aminoacids. On the other hand, removal of nutrients from the culture medium has no gross effect on cell energy balance, based on ATP measurements displayed in figure 1 D. We therefore favor the idea that a fine signaling mechanism, sensitive to physiological levels of both nutrients (glucose and aminoacids) as well as to mitochondrial dysfunction [33], regulates cell survival in our experimental setting; this mechanism has been identified in the activation of mTOR and his downstream cascade.

Experiments on HUVEC cells have been performed to test the relevance of the above mechanism in a more physiological context. These experiments have confirmed that the mTOR cascade contributes to endothelial damage by the combination of excess nutrients and growth factors scarcity, although with some differences between the two cell models. In particular, base-line mortality is higher and in part nutrient-insensitive in endothelial cells, and, as a consequence, effects of mTOR blockade on cell survival less dramatic. Conversely, drastic changes in GlcNAcylated protein accumulation in response to ambient glucose or mTOR functional status have been difficult to demonstrate in *Phoenix* cells (not shown). Notwithstanding these incongruencies, studies on endothelial cells strengthen, on one side, the role of mTOR in glycotoxicity, and underscore, on the other side, the potential of the *Phoenix* cell model in recapitulating important biochemical aspects of nutrient-related human pathology.

The downstream molecular events linking inhibition of mTOR (TORC1) to cell survival in the presented cell models needs further investigation. Although evidence of increased AkT phosphorylation/activation in cells deprived of nutrients or subdued to mTOR blockade represented an attractive candidate mechanism, our findings in *Phoenix* cells did not support this conclusion. In fact, a) cell survival by nutrient deprivation was not reverted by the PI3K inhibitor LY294002, and conversely, b) cell death in the presence of nutrients was actually attenuated by AkT/PKB blockade, while overexpression of AkT slightly decreased rescue by glutamine deprivation. Instead, since protection by Ly294002 in nutrient-rich medium (Figures 5B, a and S2) occurred in parallel with inactivation of the mTOR cascade (Figure 5B, b), these findings reinforce the idea that 1) mTOR signaling is absolutely critical for cell death in this experimental context, and 2) that beneficial effect of mTOR occurs also in the context of nearly complete AkT inhibition.

Interestingly, the detrimental action of the PI3K/AkT cascade on *Phoenix* cell survival, as suggested by data in figure 5 and S2, while rather unusual for a cancer cell line, is instead reminiscent of genetic evidence from model organisms, whereby PI3K inhibition promotes resistance to stress and longevity [34].

Other mechanisms for the protective effect of mTOR inhibition can be envisaged and deserve experimental verification.

First, inhibition of mTOR may protect cells by arresting cell cycle and preventing inappropriate G1/S transition, in the absence of growth/survival factors. Growth curves displayed in figure 1 B showing reduced but not arrested proliferation by nutrient restriction, partially support this possibility. P53, which is involved in a metabolic checkpoint induced by cell energy depletion [35], unlikely participates in cell cycle regulation in our model, since this tumor suppressor protein is functionally inactivated in *Phoenix* cells by the large T antigen. Instead, another metabolic checkpoint triggered by mitochondrial damage and accumulation of oxygen radicals, recently  described in *Drosophila* [36], is compatible with our finding of increased ROS in glucose- deprived *Phoenix* cells (Figure 2).

Second, attenuation of ER stress [37], and induction of autophagy [38] may also contribute to cell protection by inhibition of the mTOR cascade in our cellular models. In fact, of the few reported examples of mTOR pro-apoptotic activity, most refer to conditions in which ER stress can be demonstrated or at least suspected [29-31, 37]. Along similar lines, autophagy exerts important antiageing effects in model organisms and prevents cell damage by accumulation of misfolded proteins or damaged mitochondria [28]. Future work along the lines above outlined is therefore warranted.

Likewise, further effort is required to validate the above described, mTOR dependent circuitry of metabolic toxicity in tissues directly involved in nutrient-related pathology. While initial experiments performed on endothelial cells encouragingly point to this direction, (Figure 6), peripheral nerves and pancreatic beta cells definitely deserve to be investigated.

In conclusion, we have presented novel evidence for a negative regulation of cell survival by excess nutrients through the mTOR pathway. If confirmed, and extended, these observations may have important theorethical implications for the molecular under-standing of the ageing process, and significant impact on the prevention and treatment of important nutrient- and aging-associated diseases like type II diabetes and its complications.

Additionally, we have shown that HEK-293T *Phoenix* cells, an easy-to-handle and highly genetically manipulable cell line, can represent a valuable tool for mechanistic studies and pharmacological screenings related to nutrient-dependent cell damage, and by extension to stem cell biology and ageing.

## Methods

Reagents, antibodies, plasmids and cell lines. Chemicals were purchased from Sigma-Aldrich (Milan, Italy) unless differently stated. Rapamycin was from LC Laboratories (Woburn, MA), LY294002 from Cayman Chemical Company (Ann Arbor, MI). The redox-sensitive dye H2-Dichlorofluorescein Diacetate (H2-DCF-DA) was obtained from Invitrogen s.r.l. (San Giuliano Milanese, Italy).

The following primary antibodies were used: anti sir2/Sirt1 (rabbit polyclonal, cat.# 09-844) and anti SOD2 (rabbit polyclonal, cat.# 06-984) from Upstate Biotechnology/Millipore (Vimodrone, Milan, Italy); anti-actin (goat polyclonal, cat #sc-1615 and sc-1616), anti S6 kinase 1 (rabbit polyclonal, C18, sc-230), and anti mTOR/FRAP (rabbit polyclonal, C19-R, cat.# sc-1550-R) from Santa Cruz Biotechnology Inc. (Heidelberg, Germany); anti p-S6K1, Thr 389 (cat# 9205); anti p-S6, Ser 235-236 (cat#2211); anti p-4EBP1, Thr 37-46, (cat# 2855P); anti p-AMPK α, Thr 172, (cat# 2531); anti phosho-(Ser 437) AkT, (cat# 9271); anti AkT, (cat# 9272); anti p-GSK3-β, Ser-9, (cat# 9336), all from Cell Signaling Technology (Danvers, MA). HRP-conjugated goat anti rabbit IgG antiserum was from BIORAD (Segrate, Milan, Italy).

The plasmid encoding the human Sirt-1 cDNA in the pBabe Puro vector backbone was kindly provided by Dr. Michael Greenberg (Harvard Medical School, Boston, MA). Expression constructs for rat mTOR (pcDNA3 vector, Invitrogen) and human Catalase (pLNCX vector, Clontech, Mountain View, CA) were a gift of Dr. Toren Finkel (NHLBI, NIH, Bethesda, MD). The construct encoding a myristoylated, constitutively active mutant of human AkT fused to the Estrogen Receptor ligand binding domain (Myr(Δ1-129)-AkT-HA-ER) in the pWZL-hygro retroviral vector [27] was provided by Dr. Barbara Bedogni (University of Stanford, CA).

The pcDNA3-based construct encoding human SOD2 was described elsewhere [39]. Mission™ shRNA clones constructed within the lentivirus plasmidvector pLKO.1-Puro were purchased from Sigma Aldrich (Milan, Italy).

293-T *Phoenix *cells, a retrovirus packaging line derived from E1A-transformed embryonic human kidney cells (HEK-293) carrying a temperature sensitive T antigen [40], were kindly provided by Dr. G. Nolan (University of Stanford, CA). A detailed description of this cell derivative can be found in the Nolan's Laboratory Home Page (http://www.stanford.edu/group/nolan/retroviral_systems/phx.html).

Cells were routinely maintained in Dulbecco's Modified Eagle's Medium (DMEM) containing 4.5 g/l glucose, 2 mM Glutamine, 1 mM Sodium Pyruvate, Non Essential Aminoacids and Penicillin-Streptomycin (EUROBIO, Les Ulis, France).

Human Umbilical Vein Endothelial Cells (HUVEC) were obtained from Lonza/CloneticsÒ (Walkersville, MD, USA) and maintained in EBM-2 Basal Medium supplemented with hEGF, VEGF, B-FGF, IGF-1, Hydrocortisone, Heparin, Acorbic Acid, Gentamicin, Amphotericin B and 2% FBS (EGM-2 Bulletkit, Lonza, CC-3162). For experimental procedures cells between passages 4 and 7 were used.

Cell viability assay. *Phoenix* Cells were seeded at 10^5 ^cells/well, in 24-well plate in complete medium and incubated for 16 to 24 hours.Medium was then replaced with glucose-free/glutamine-free DMEM (Eurobio) (basic formulation as reported in supplemental table 1), 1 mM Pyruvate, Penicillin-Streptomycin and 1 mM HEPES pH 7.4. When necessary the medium was supplemented with serum (or Bovine Serum Albumin, BSA), glucose, glutamine and Non Essential Aminoacids (NEAA, formulation of the 50X solution in supplemental table 2). Pharmacological inhibitors were also added at this stage, at the following concentrations: 2-deoxyglucose (2-DG), 10 mM; Rapamycin, 200 nM; Ly294002, 20 μM; Rotenone, 5 μM; 3-nitropropionic acid (3-NPA), 1 mM; 2,4 Dinitrophenol, DNP, 1 mM; N-Acetyl Cysteine (NAC) 10 mM. After 72-96 hours incubation (humidified incubator, 37°C, 5% CO_2_) live and dead cells were collected by gentle pipetting and transferred into dedicated vials for flow cytometry (COULTER Epics, 480 nm Argon laser lamp). Immediately before analysis Propidium Iodide was added at 1 μg/ml. PI-positive cells (FL-3) were scored as dead cells after threshold definition with unstained cells; cell debris was gated out and excluded from the analysis [41].

HUVEC cells were seeded in 12 well plates at 10^5^ cells/well in complete medium, and left to adhere for 12 hours. Medium was then replaced with glucose-free DMEM containing 5% FBS but no specific endothelial growth factor, and D-glucose was added from a 300 mM stock (in PBS) at the desired dilution. Rapamycin was added at 200 nM at this stage and 24 hours later, without medium change.

After 48-60 hours of incubation cells were trypsinized, pooled with floating cells and analysed by flow cytometry, as described in reference 21. Live and dead cells were identified based on the position on the forward scatter-side scatter plot, and the % of cell survival calculated by the formula live cells/(live cells+dead cells).

Cell proliferation assay. Cells were seeded at 10^4^ cells/well, in 24-well plate in complete medium. 16-24 hours later medium was replaced with glucose or glutamine-free DMEM without FBS. Live cells from triplicate wells were counted at different time-points (0, 24, 48 and 72 hours) by an hemocytometer; dead cells were excluded based on morphology and trypan blue uptake.

Cell transfections. Transient transfections of *Phoenix* cells were made with the EFFECTENE reagent (QIAGEN, Hilden, Germany), directly in 24 well plate, using about 150 nanograms DNA/well. A master transfection mix for 6 wells typically contained 1 μg DNA, 4 μl of Enhancer and 10 μl of transfection reagent, according to the manufacturer's indications with minor changes. Transfection efficiency was routinely above 50% in these conditions, based on flow cytometry of cells transfected with a GFP expressing plasmid. After 24-36 hours cells were used for survival assay or biochemical analysis (see below).

Lentiviral-mediated RNA interference. Recombinant vesicular stomatitis virus(VSV)-pseudotyped lentiviral vectors were obtained by standard procedure, according to Tiscornia et al. [42]. Briefly, 293 Thuman embryonic kidney cells were co-transfected by calcium phosphate with the lentiviral packaging(pMDLg/RRE), envelope (pMD2.G), and rev-expressing (pRSV-REV) plasmids, together with the pLKO.1-based short hairpin constructs specific for ***mTOR*** (TRCN0000038677) or the Mission™ non-target control vector. Viral supernatants were collected 48 hours aftertransfection, filtered through 0.22-μm pore nitrocellulosefilters, concentrated by ultracentrifugation at 50,000 x *g* for140 min at RT and stored at -80 C until use. Target cells were transduced with the lentiviral vector stocks in presence of 6 μg/ml Polybrene and selected using puromycin-containing medium.

Confocal analysis of cell oxidation and intracellular NAD(P)H. Cells were seeded in 35 mm glass bottom dishes (Ibidi, Integrated Biodiagnostic, Martinsried, Germany) and transfected with 0.75 μg of a construct encoding the redox-sensitive Yellow Fluorescent Protein (mt-rxYFP). After 48 hours cells were deprived of serum and nutrients for additional 24 hours, and fluorescent cells imaged and quantified by confocal microscopy (Leica, DM-IRE2 Germany) as described in detail elsewhere [43]. Briefly, fluorescence signals of samples excited at 488 nm (F_488_) and at 458 nm (F_458_) were measured and the ratio (**F** = F_488_/F_458_) calculated. Values of F for completely reduced (F_red_) and completely oxidised (F_ox_) rxYFP were obtained from literature [44]. Pseudocolor images were constructed based on ***R*** values, defined as

### *R*= (F-F_ox_)/(F_red_-F_ox_)

and ranging between 0 (complete oxidation) and 1 (complete reduction), by means of a dedicated software generated through the Labview 7.1 interface [23]. For image quantitation, average *R* values were determined within multiple Regions of Interest (ROIs, single cells or small cell clusters) for each sample, and their Mean±SD (n = 7 to 9) determined and utilized for further statistical analysis (Student *t-*test). In some experiments, after initial cell imaging in nutrient-deficient medium, nutrients were added back and cell redox responses monitored for 30 minutes or longer.

Intracellular NAD(P)H was measured, in the same experimental settings as above, by two-photon confocal analysis of cell green autofluorescence after two-photon excitation at 366 nm, as described by Patterson et al. [44].

Biochemical studies. For protein phosphorylation studies *Phoenix* cells were plated at 1.5 x 10^5^/well in 12-well plate and incubated for 16 to 24 hours in complete medium. The day after, cells were switched to DMEM without glucose, glutamine/NEAA and FCS, and these components were added back where necessary. Serum-free samples were given BSA 20 mg/ml (stock) at the same dilution as FCS (typically 10%, i.e. 2 mg/ml final concentration). Antioxidants and chemical inhibitors were also added at this stage of the experiment (see above, viability assay). After 24 hours supernatants were removed and cells lysed in 100 microliters of ice-cold lysis buffer (NaCl 150 mM, Tris-hcl 50 mM pH 8; 2 mM EDTA) containing 1% v/v Triton X-100, 0.1 % v/v SDS, 1:1000 Protease Inhibitor cocktail (Sigma), 1 mM Sodium Orthovanadate, 1 mM NaF; 2 mM β-glycerophosphate. After 15 minutes on ice with occasional vortexing cells were spun down at 14,000 rpm, 4°C to remove debris and unlysed cells, and supernatant quantified for protein content (DC Protein Assay, BIORAD), resuspended in 6X Laemmli buffer, boiled for 2 minutes and stored at -80°C or directly loaded onto denaturing discontinuous polyacrylamide gels for SDS-PAGE. Proteins were then electroblotted onto nitrocellulose membrane (PROTRAN®, Whatman, Dassel, Germany). After reversible Ponceau S staining to confirm protein transfer and equal loading throughout the lanes, membranes were blocked in TBS-T containing 5% skim milk. Antisera were added in 3% milk at the appropriate dilution and incubated for 16 hours on a rotating plate at 4°C. After extensive wash in TBS-T, immuno-complexes were visualized by incubation with HRP-conjugated secondary reagents (BIORAD) followed by enhanced chemoluminescence (ECL, GE Healthcare, Milan, Italy) and autoradiography. In some experiments autoradiograms were digitalized and band intensity (band volume, i.e. area x mean pixel intensity) quantified with a dedicated software (Quantity One, BIORAD). Quantitation was normally not performed when differences displayed were immediately evident. Occasionally membranes were stripped in 2% SDS at 60°C, washed, blocked and subdued to a second round of hybridization.

For protein O-glycation and phosphorylation studies on HUVEC, cells were handled as for viability assays, except that after 24 hours of incubation supernatants and floating cells were removed and adherent cells lysed as described above.

Accumulation of GlcNAcylated proteins was determined by immunoblotting using a specific anti O-GlcNAc monoclonal antibody (CTD 110.6, COVANCE [45]).

Catalase assay. As an indirect assessment of intracellular catalase activity, cells were switched to serum-free medium (without BSA), loaded for 30 minutes with the redox sensitive fluorescent dye H2-Dichlorofluorescein Diacetate (H2-DCF-DA) and challenged with 1 mM hydrogen peroxide for 15 minutes. Cells were then quickly transferred to tubes for flow cytometry and green fluorescence (Fl-1) quantified. Resistance to H_2_O_2_-induced cell oxidation was assumed to correlate with cell capacity to degrade hydrogen peroxide. Extracellular catalase and the catalase inhibitor Aminotriazol were used to validate this procedure.

Determination of intracellular ATP . ATP was quantified by chemiluminescence using a dedicated kit (ENLITEN^®^ ATP Assay, PROMEGA, Milan, Italy) according to the manufacturer's recommendations. For each sample luminescent emission was normalized for total protein content, determined as described above.

Statistics. Data sets (usually triplicate culture wells) were compared by the two-tailed Student's *t*-test for independent samples. Threshold for statistical significance was set at *p*<0.05

## Supplementary data

Supplementary Figure 1 Inhibition of AkT phosphorylation by mTOR re-expression in sh-TOR Phoenix cells. Cells were analyzed as in figure 5A, after 24 hours of serum starvation, in the presence of nutrients. Densitometry of the phospho (Ser 308) AkT band is reported. Picture representative of two independent experiments.

Supplementary Figure 2 A constitutively active mutant of AkT (myrAkT-ER) fails to protect Phoenix cells from serum starvation and high nutrients. ***a*** Survival assay displaying a slight increase in mortality of glutamine-deprived cells expressing the myrAkT-ER mutant. All cultures were exposed to 1 mM 4-hydroxy-Tamoxifen (4-OHT) for the entire period of incubation (72 hours); note that transfection efficiency was 50% at most in this and other experiments. Values are Mean SD of triplicate samples. Significance was determined by unpaired, two-tailed Student t-test. Representative of two experiments with two independent transfections. ***b*** Western blot analysis confirming expression, responsiveness to 4-OHT and activity of the myrAkT mutant in cells grown in standard medium containing FCS. myrAkT-ER accumulates in response to 4-OHT as revealed by anti-tag (HA) immunoblot. Phosphorylation of the AkT substrate GSK3-β on Serine 9 was evaluated as an index of AkT activity (lower panel). Equal protein loading was confirmed by anti actin immunoblot (middle panel). Representative of two independent experiments.

Supplementary Table 1 Formulation of Glucose-free/Glutamine-free DMEM.

Supplementary Table 2 Formulation of the DMEM Non essential aminoacids supplement solution (50x).
