# Supplementary figures and images for: Nutrient withdrawal rescues growth factor-deprived cells from mTOR-dependent damage
Source: Aging (Albany NY). 2010 Aug 24;2(8):487–503. doi: 10.18632/aging.100183 (PMC2954040; doi:10.18632/aging.100183)

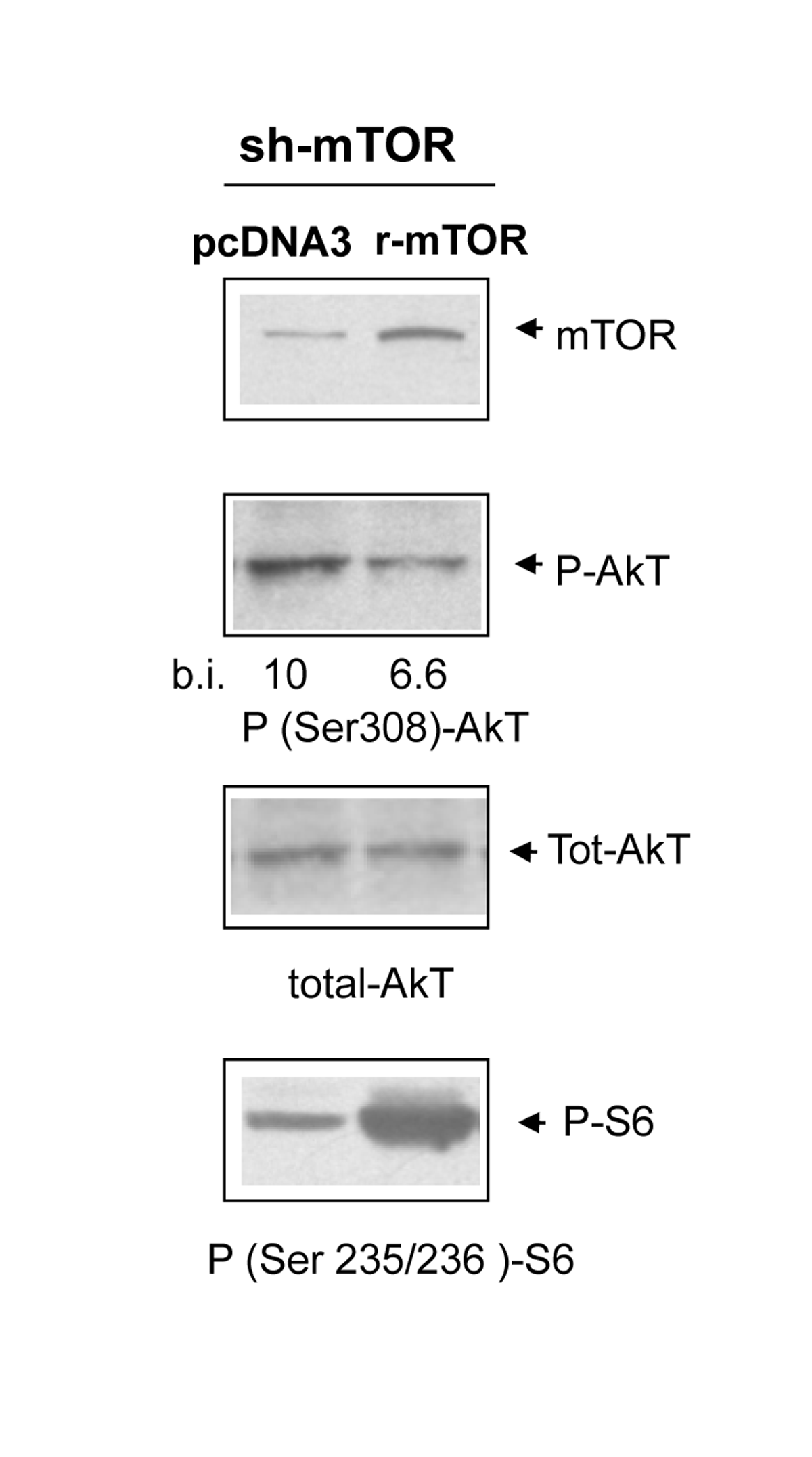

Supplement: Supplementary Figure 1 — Inhibition of AkT phosphorylation by mTOR re-expression in sh-TOR Phoenix cells. Cells were analyzed as in figure 5A, after 24 hours of serum starvation, in the presence of nutrients. Densitometry of the phospho (Ser 308) AkT band is reported. Picture representative of two independent experiments. [file aging-02-487-s001.tif]

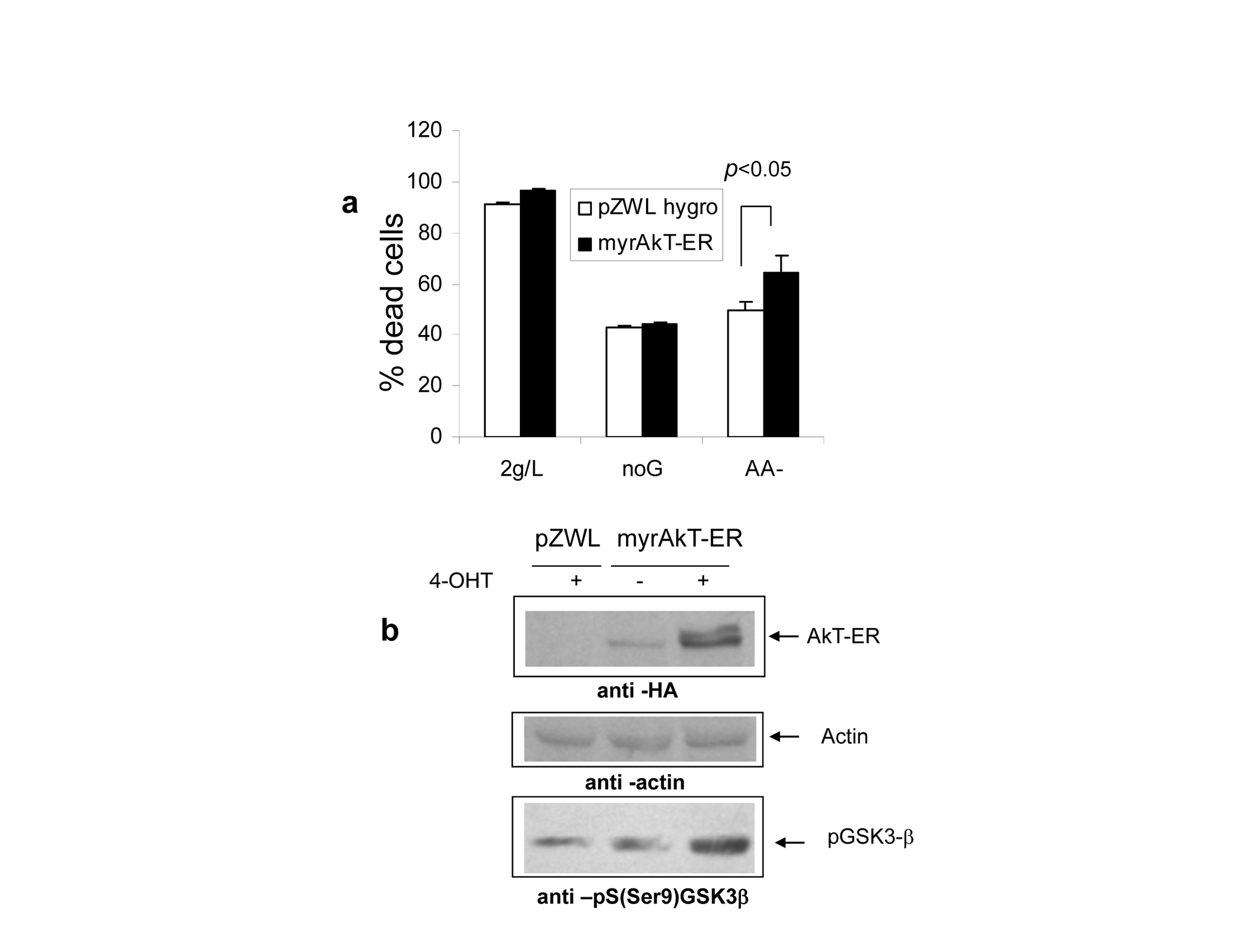

Supplement: Supplementary Figure 2 — A constitutively active mutant of AkT (myrAkT-ER) fails to protect Phoenix cells from serum starvation and high nutrients. a Survival assay displaying a slight increase in mortality of glutamine-deprived cells expressing the myrAkT-ER mutant. All cultures were exposed to 1 mM 4-hydroxy-Tamoxifen (4-OHT) for the entire period of incubation (72 hours); note that transfection efficiency was 50% at most in this and other experiments. Values are Mean SD of triplicate samples. Significance was determined by unpaired, two-tailed Student t-test. Representative of two experiments with two independent transfections. b Western blot analysis confirming expression, responsiveness to 4-OHT and activity of the myrAkT mutant in cells grown in standard medium containing FCS. myrAkT-ER accumulates in response to 4-OHT as revealed by anti-tag (HA) immunoblot. Phosphorylation of the AkT substrate GSK3-β on Serine 9 was evaluated as an index of AkT activity (lower panel). Equal protein loading was confirmed by anti actin immunoblot (middle panel). Representative of two independent experiments. [file aging-02-487-s002.tif]
