# Supplementary material for: Nutrient withdrawal rescues growth factor-deprived cells from mTOR-dependent damage
Source: Aging (Albany NY). 2010 Aug 24;2(8):487–503. doi: 10.18632/aging.100183 (PMC2954040; doi:10.18632/aging.100183)
Supplement: Supplementary Table 1 [file aging-02-487-s001.doc]

**Supplementary Table 1**

Formulation of Glucose-free/Glutamine-free DMEM

| **Inorganic Salts** | **mg/L** |
| --- | --- |
| Calcium Chloride anhydrous | 200.00 |
| Ferric(III)-Nitrate•9H2O | 0.10 |
| Potassium Chloride | 400.00 |
| Magnesium Sulphate anhydrous | 97.70 |
| Sodium Chloride | 6400.00 |
| Sodium Dihydrogen Phosphate• H2O | 125.00 |
| Carbonate | 3700.00 |
| **Amino Acids** |  |
| L-Arginine• HCl | 84.00 |
| L-Cystine | 48.00 |
| L-Glutamine | 0 |
| Glycine | 30.00 |
| L-Histidine• HCl• H2O | 42.00 |
| L-Isoleucine | 105.00 |
| L-Leucine | 105.00 |
| L-Lysine• HCl | 146.00 |
| L-Methionine | 30.00 |
| L-Phenylalanine | 66.00 |
| L-Serine | 42.00 |
| L-Threonine | 95.00 |
| L-Tryptophan | 16.00 |
| L-Tyrosine | 72.00 |
| L-Valine | 94.00 |
| **Vitamins** |  |
| D-Calcium-Pantothenate | 4.00 |
| Choline Chloride | 4.00 |
| Folic Acid | 4.00 |
| Myo-Inositol | 7.20 |
| Nicotinamide | 4.00 |
| Pyridoxal• HCl | 4.00 |
| Riboflavin | 0.40 |
| Thiamine• HCl | 4.00 |
| **Other Components** |  |
| D-Glucose | 0 |
| Phenol Red | 15.00 |
| Sodium Pyruvate | 0 |
