# Supplementary material for: Nutrient withdrawal rescues growth factor-deprived cells from mTOR-dependent damage
Source: Aging (Albany NY). 2010 Aug 24;2(8):487–503. doi: 10.18632/aging.100183 (PMC2954040; doi:10.18632/aging.100183)
Supplement: Supplementary Table 2 [file aging-02-487-s002.doc]

**Supplementary Table 2.**

Formulation of the DMEM Non Essential Aminoacids Supplement solution (50x)

|  | **mg/L (50x)** |
| --- | --- |
| L-Arginine• HCl | 6320.00 |
| L-Cystine | 1201.00 |
| L-Histidine• HCl• H2O | 2096.00 |
| L-Isoleucine | 2623.00 |
| L-Leucine | 2623.00 |
| L-Lysine• HCl | 3625.00 |
| L-Methionine | 755.00 |
| L-Phenylalanine | 1651.00 |
| L-Threonine | 2382.00 |
| L-Tryptophan | 510.00 |
| L-Tyrosine | 1811.00 |
| L-Valine | 2343.00 |
